# Relationship between ankle strength and range of motion and postural stability during single-leg quiet stance in trained athletes

**DOI:** 10.1038/s41598-021-91337-6

**Published:** 2021-06-03

**Authors:** Nebojša Trajković, Žiga Kozinc, Darjan Smajla, Nejc Šarabon

**Affiliations:** 1 Faculty of Sport and Physical Education, University of Niš, 18000 Niš, Serbia; 2 Faculty of Health Sciences, University of Primorska, Polje 42, 6310 Izola, Slovenia; 3 Andrej Marušič Institute, University of Primorska, Muzejski trg 2, 6000 Koper, Slovenia; 4 Human Health Department, InnoRenew CoE, Livade 6, 6310 Izola, Slovenia; 5 Laboratory for Motor Control and Motor Behavior, S2P, Science to Practice, Ltd., Tehnološki Park 19, 1000 Ljubljana, Slovenia

**Keywords:** Anatomy, Medical research, Risk factors

## Abstract

The aim of this study was to determine the relationship between strength of ankle plantar and dorsal flexors and range of motion (RoM), and body sway variables during single-leg quiet stance, in highly trained athletes. The participants for this study were young athletes from 9 disciplines (n = 655). Center of pressure (CoP) velocity, amplitude, and frequency were measured during single-leg quiet stance. Moreover, athletes were measured for passive ankle plantar flexion (PF) and dorsal flexion (DF) RoM, and for rate of torque development (RTD) in the 0–50 (RTD50) and 0–200 ms time windows (RTD200). Ankle strength and RoM could not predict CoP velocity total, anterior–posterior (AP), and medial–lateral (ML) (p > 0.05). However, PF_RTD50_ and PF_RoM_ and PF_RoM_ positively influenced CoP amplitude in ML direction (p < 0.001, R^2^ = 0.10). Moreover, CoP frequency in ML direction significantly increased with lower PF_RTD50_, DF_RTD50_, DF_RTD200_, PF_RoM_, and DF_RoM_ (p < 0.05). We have demonstrated that ankle strength and RoM were related to single-leg quiet stance postural balance in trained athletes. The ankle RoM showed the greatest influence on CoP variables in ML directions.

## Introduction

Postural balance is the ability to maintain control of the body position above the surface of the support^1^ which is necessary during the performance of various activities of daily living. It is also one the most important skills that protect athletes from injury. This was confirmed in several studies that showed athletes with higher CoP displacements are more prone to injuries in the subsequent training period^2–4^. Moreover, larger postural sway was associated with lower incidence of lower limb injuries in prospective study conducted on soccer players^5^. The study of postural control in larger group of athletes from different sport could provide some insight into the development of specific postural strategies required by a particular sport.

Several tests for assessing static balance are used routinely in sport and clinical practice, from simple field tests, to instrumented laboratory tests. The most reliable way to evaluate static balance is the recording of center of pressure (CoP) movements during quiet standing, which can be done using a force plate^6^. Postural sway analysis is performed by processing CoP time series. CoP sway variables are commonly analyzed in a direction specific manner, meaning that the signal is decomposed to AP (anterior–posterior) and ML (medial–lateral) directions^7^. While bipedal quiet stance has been the most common condition used to assess postural sway, according to Riemann, and Schmitz^8^, unipedal stance has more applications in clinical and sports medicine settings. Moreover, Nevitt et al.^9^ found that most injurious falls occurred in activities that involved single-leg stance.

It was reported that athletes from different sports have reduced sway velocity and sway amplitude compared to non-trained subjects^10–13^. Furthermore, high-level athletes exhibit less sway in contrast to low-level athletes^14^. Previous studies have tried to identify different factors that influence postural sway, such as visual information^15^, feet position^16^, motor imagery^17^, light touch^18^, age^19^, and daily life physical activity^14,20^. Nevertheless, the influence of other factors remains unknown. Most of the studies that examined the impact of strength and RoM on postural stability were conducted in older adults^21–23^. One recent study^24^ carried out on young adults investigated the relationship between bipedal quiet stance postural sway (CoP length and velocity), ankle RoM, and lower-extremity muscle strength. The results revealed that postural sway was negatively influenced by muscle strength of plantar flexors (PF) and (dorsal flexors) DF, and ankle PF RoM. Another study showed that low ROM of PF was negative related to overall balance in students aged 20 years^25^.

Understating the mechanisms of postural control in highly trained athletes at different sporting levels is of paramount importance for reducing the risk of injuries and the optimization of the training process^26,27^. There is a strong evidence that elite athletes from different sport disciplines show difference in postural control^28^. Therefore, there is an urgent call for similar studies with larger sample sizes, using different body sway variables. Additionally, since most of the studies have used bipedal stance for CoP movements, it would be interesting to investigate the relationship between ankle strength and single-leg quiet stance body sway in young highly trained athletes. Moreover, there is a lack of studies that examined the associations between RoM and body sway during single-leg quiet standing. Therefore, the aim of this study was to determine the relationship between ankle strength and RoM and the characteristics of single-leg body sway in highly trained athletes. We hypothesized that a strength, and RoM ankle outcome measures would be significant predictors of AP and ML postural stability during single-leg stance.

## Methods

### Participants

The participants for this study were young athletes from 9 disciplines (n = 655; age range = 13–33 years). The details regarding the sample sizes of individual groups are available in Table 1, alongside basic demographic data. The exclusion criteria were any lower leg injuries in the last 6 months and any self-reported neurological or non-communicable diseases. The inclusion criteria included at least 8 h of sports practice per week; more than 3 years of sports practice for younger athletes, and more than 5 years for young adults. Moreover, the athletes were members of teams in selected sports disciplines that compete on national level or participants of national and international sports competitions. The participants were informed about the testing procedures in detail, and were requested to sign a written informed consent prior to the measurements. For underage participants, their parents or legal guardians were also informed and signed the informed consent on their behalf. The experimental protocol was approved by Republic of Slovenia National Medical Ethics Committee (approval no. 0120-99/2018/5) and was performed in accordance to the latest revision of the Declaration of Helsinki.

**Table 1:** Athlete characteristics (values are presented as means ± standard deviation).

	N (female)	Age (years)	Body height (cm)	Body mass (kg)	BMI (kg/m^2^)
Basketball	165 (58)	16.7 ± 1.3	183.9 ± 9.8	75.6 ± 12.9	22.2 ± 2.63
Dance	77 (54)	22.6 ± 6.6	170.3 ± 7.5	60.0 ± 9.7	20.5 ± 2.1
Soccer	169	17.4 ± 3.4	179.4 ± 7.1	70.2 ± 9.5	21.7 ± 2.2
Track and field	29 (8)	17.8 ± 2.7	176.8 ± 8.0	70.1 ± 9.5	22.3 ± 1.9
Volleyball	42	16.9 ± 3.8	183.2 ± 9.3	73.4 ± 12.7	21.7 ± 2.4
Alpine skiing	9	22.7 ± 3.3	181.5 ± 6.9	82.3 ± 5.6	24.9 ± 0.9
Tennis	110 (42)	16.9 ± 10.4	172.7 ± 11.0	62.2 ± 12.8	20.6 ± 2.5
Martial arts	35 (17)	18.7 ± 4.9	172.5 ± 9.5	68.7 ± 16.0	22.8 ± 3.3
Speed skating	19 (7)	16.6 ± 4.3	166.5 ± 13.8	59.5 ± 14.9	21.1 ± 3.0

### Experimental procedures

We used an isometric dynamometer (S2P, Science to Practice, Ljubljana, Slovenia) to assess ankle strength (force sensor: model 1-Z6FC3/200 kg, HBM, Darmstadt, Germany). The participant’s shins were tightly secured within the dynamometer, and the feet were placed on a rigid plate mounted above a torque sensor. The sensor built into the dynamometer was embedded in a way that made it function as a torque sensing device. Namely, the force sensor was positioned on a fixed length lever arm in relation to the axis. The axis of the dynamometer was aligned with the medial malleolus. The ankle was in a neutral position (90°), which was achieved by adjusting seat height and seat depth if needed. The feet were tightly fixated against the plates with a strap. The participants were at all times provided with loud verbal encouragement and were instructed to push “as fast and as hard as possible’’ and sustain the maximal effort for ~ 3 s. Three repetitions were performed, with 1 min breaks in between. The signals were processed automatically with arithmetic mean filter (5 ms window) in the manufacturer’s software (Analysis and Reporting Software, S2P, Ljubljana, Slovenia). The peak torque (T_max_) value during each maximal voluntary contraction trial was quantified as the maximal 1-s mean value of the trial. Next, the rate of torque development (RTD) was calculated as Δ torque/Δ time for 0–50 (RTD50) and 0–200 ms (RTD200) time windows after the onset of the contraction. The onset of torque rise was automatically detected at the instant at which the baseline signal exceeded the 3% of the peak torque. The maximal values from the three repetitions were taken for further analysis.

Passive ankle RoM PF and DF were assessed with participants lying supine, with the foot and bottom part of the shin over the edge of the table. A small towel was put under the shin at the edge of the table. One examiner moved the ankle joint into maximal PF and DF (grasping the foot at the base of the toes) with one hand, while stabilizing the shin with the other hand. The second examiner assessed the RoM with a goniometer, aligning the axis with the medial malleolus, with the immovable arm aligned with the shin and the movable arm pointing to the base of the thumb. Three repetitions were performed for each movement, and the mean value was taken for further analyses.

The body sway was assessed in a single-leg stance without footwear. The participants were instructed to stand as still as possible on the force platform and to look at a fixed point (black dot on a white background, at an approximately eye level and ∼ 4 m away from the participant). Participants performed three 30-s repetitions with each leg and 60-s breaks were provided between repetitions. For each repetition, the participants acquired the single-leg position, and the examiner started the acquisition after they had stabilized (1–2 s). Both legs were examined in an alternating order across repetitions, with the starting leg being randomized for each participant. The hip of the opposite (i.e., non-standing) leg was in a neutral position (0°), the thigh was parallel to the standing leg, while the knee was flexed at 90° and was not allowed to touch the standing leg. The knee of the standing leg was in the extended position but not hyperextended (locked). The hands had to be placed on the hips. A piezoelectric force platform (model 9260AA, Kistler, Winterthur, Switzerland) was used to collect the ground reaction force data at a sampling rate of 1000 Hz. The data was automatically filtered (low-pass Butterworth, 2nd order, 10 Hz) in the MARS Software (version 4.0, Kistler, Winterthur, Switzerland). The data was further automatically processed in MARS to obtain the outcome measures of interest. For all the outcome variables, the average of the three repetitions was used for further analyses. We considered the mean CoP velocity (total, AP and ML), CoP amplitude (AP and ML) and CoP frequency (AP and ML). The CoP amplitude was determined as the average CoP sway in AP or ML direction, calculated as the common length of the trajectory of the COP sway only in the given direction, divided by the number of changes of movement direction. The CoP frequency was defined as the frequency of the oscillations of the CoP calculated as the number of peaks in AP or ML direction (i.e. changes in the direction of CoP movement) divided by the measurement time^29^.

### Statistical analysis

Data were analyzed using SPSS (version 20.0, SPSS Inc., IBM company, Armonk, NY, USA) and presented as mean ± standard deviation unless otherwise stated. Normality of data was assessed and verified by Kolmogorov–Smirnov test. An independent T-test estimated the mean differences (95% confidence interval) between men and women in the outcome variables (CoP velocity, amplitude, and frequency) and the predictors (PF and DF strength and RoM measures). We used a T-test that is robust for unequal variances with a Satterthwaite approximation in case the Levene’s test yielded unequal variances. A correlation matrix of pairwise Pearson’s correlation coefficient (r) assessed the bivariate linear relationships between outcomes (CoP velocity: total, AP, ML; CoP amplitude: AP, ML; and CoP frequency: AP, ML) and predictors (gender, age, body mass index (BMI), PF and DF MVCt, RTD 0–50 ms and 0–200 ms and RoM measures) prior study hypotheses testing. Pearson’s r values of ± 0.10, ± 0.30, and ± 0.50 demarcated the weak, moderate, and strong relationships^30^.

We used a hierarchical model building to explore the relationship between CoP velocity (total, AP, ML), CoP amplitude (AP, ML), and CoP frequency (AP, ML) and DF and PF strength (MVCt, RTD 0–50 ms and 0–200 ms) and DF and PF RoM (Block 2) while controlling for age, gender, and BMI effects (Block 1). The Kolmogorov–Smirnov test and visual inspection of a residual scatter plot confirmed normality and homogeneity of residuals, respectively. Sequential linear regressions analysis was adopted for each outcome. No presentence of multicollinearity was registered in each model according to the variance inflation factor (VIF < 10). We reported standardized coefficients and respecting p values from each model to depict relationships between predictors and each outcome, and to illustrate relative contribution of each predictor to the outcome. The coefficient of determination (R2) adjusted for the number of predictors in the model (adjusted R2) are reported as the measures of goodness of fit of each model. The change in R2 and F from Block 1 to 2 illustrated overall fit of the models for each outcome only in the function of DF and PF strength and RoM measures. The significance was set at p ≤ 0.05.

## Results

Descriptive data for ankle muscle strength and RoM and CoP variables are presented in Table 2.

**Table 2:** Descriptive data for ankle PF and DF muscle strength, RoM and CoP variables.

	Basketball	Dance	Soccer	Track and field	Volleyball	Alpine skiing	Tennis	Martial arts	Speed skating
**CoP velocity (mm/s)**									
Total	45.1 ± 13.1	35.3 ± 7.8	45.3 ± 10.6	48.1 ± 13.0	40.6 ± 8.9	51.4 ± 9.7	43.7 ± 9.0	39.4 ± 11.1	42.0 ± 11.3
AP	28.3 ± 8.9	20.3 ± 5.3	28.1 ± 7.6	27.3 ± 7.6	24.8 ± 6.7	28.8 ± 4.5	26.8 ± 6.1	22.2 ± 6.8	23.5 ± 7.6
ML	29.5 ± 8.6	24.6 ± 5.5	29.7 ± 6.8	33.8 ± 10.1	26.9 ± 5.6	36.6 ± 9.4	28.9 ± 6.1	28.0 ± 8.0	30.0 ± 7.9
**CoP amplitude (mm)**									
AP	6.4 ± 2.2	4.4 ± 1.2	6.0 ± 1.9	5.5 ± 1.4	5.6 ± 1.7	6.4 ± 1.4	6.0 ± 1.5	5.0 ± 1.9	4.4 ± 1.9
ML	7.0 ± 2.7	4.2 ± 1.6	6.4 ± 2.3	4.7 ± 2.6	5.3 ± 2.0	5.7 ± 2.9	8.3 ± 2.2	5.8 ± 2.3	3.8 ± 1.7
**CoP frequency (Hz)**									
AP	4.6 ± 0.6	4.7 ± 0.8	4.8 ± 0.7	5.0 ± 0.6	4.5 ± 0.6	4.6 ± 0.6	4.5 ± 0.6	4.7 ± 0.8	5.9 ± 2.1
ML	4.5 ± 0.9	6.8 ± 3.1	5.0 ± 1.4	8.0 ± 2.3	5.6 ± 2.6	7.1 ± 1.4	3.7 ± 1.0	5.2 ± 1.2	8.6 ± 3.1
PF MVC_t_ (Nm)	43.5 ± 10.5	30.2 ± 8.2	49.8 ± 14.1	49.4 ± 13.0	43.0 ± 12.2	49.1 ± 7.8	34.4 ± 9.1	35.1 ± 9.0	31.0 ± 8.7
PF_RTD50_ (Nm/ms)	219.5 ± 72.1	79.7 ± 49.2	228.8 ± 119.5	191.4 ± 89.7	162.1 ± 102.1	75.2 ± 31.8	182.0 ± 72.0	101.6 ± 83.8	54.4 ± 44.1
PF_RTD200_ (Nm/ms)	172.0 ± 47.7	116.9 ± 30.8	196.5 ± 59.7	202.5 ± 60.2	165.0 ± 54.4	166.7 ± 41.6	134.8 ± 41.8	133.5 ± 45.9	108.3 ± 44.0
DF MVC_t_ (Nm)	133.4 ± 42.0	131.7 ± 30.6	152.1 ± 42.5	159.6 ± 48.0	154.2 ± 38.0	153.7 ± 2.9	116.7 ± 37.5	136.5 ± 38.2	112.5 ± 48.4
DF_RTD50_ (Nm/ms)	434.1 ± 176.8	235.2 ± 125.8	422.5 ± 198.9	412.7 ± 231.6	313.0 ± 186.4	161.2 ± 56.4	394.2 ± 177.0	390.1 ± 219.5	181.0 ± 134.7
DF_RTD200_ (Nm/ms)	478.4 ± 164.4	446.8 ± 119.3	533.4 ± 162.1	574.2 ± 202.3	531.0 ± 152.9	508.4 ± 76.7	428.8 ± 150.1	468.5 ± 145.2	354.7 ± 202.1
PF_RoM_ (°)	84.2 ± 10.9	85.2 ± 8.4	73.3 ± 8.3	71.1 ± 8.3	74.3 ± 8.0	76.3 ± 10.0	91.4 ± 8.8	78.9 ± 7.6	81.5 ± 8.4
DF_RoM_ (°)	12.2 ± 10.7	16.7 ± 7.0	17.8 ± 9.2	11.1 ± 7.6	12.5 ± 8.6	19.8 ± 7.6	17.8 ± 8.7	14.1 ± 11.4	19.9 ± 8.6

*AP* anterior–posterior, *ML* medial–lateral, *PF MVCt* maximal voluntary contraction torque for plantar flexion, *PF*_*RTD50*_ plantar flexion rate of torque development for 0–50 ms, *PF*_*RTD200*_ plantar flexion rate of torque development for 0–200 ms, *DF MVCt* maximal voluntary contraction torque for dorsal flexion, *DF*_*RTD50*_ dorsal flexion rate of torque development for 0–50 ms, *DF*_*RTD200*_ dorsal flexion rate of torque development for 0–200 ms, *PF*_*RoM*_ plantar flexion range of motion, *DF*_*RoM*_ dorsal flexion range of motion.

### Correlations

Results of the correlational analyses between PF and DF strength and RoM and CoP variables are presented in Table 3. In general, we found significant and weak correlations between variables. The ankle PF and DF strength showed a significant positive weak correlation with total, AP and ML CoP velocity (r = 0.07–0.18), and CoP amplitude (r = 0.07–0.19) and negative correlations with CoP frequency (r from − 0.06 to − 0.21). Similar results were found for the relationship of PF RoM and body sway variables with negative and weak correlations found in CoP velocity (r from − 0.07 to − 0.09) and CoP frequency (r from − 0.08 to − 0.19) and positive correlations with CoP amplitude in ML directions (r = 0.12–0.13). DF RoM was positively related to amplitude in ML directions (r = 0.12–0.17) and negatively to CoP frequency (r from − 0.15 to 0.16).

**Table 3:** Correlation matrix of Pearson’s r for body sway variables and predictors (N = 633).

Predictors	CoP velocity			CoP amplitude		CoP frequency	
	Total	AP	ML	AP	ML	AP	ML
Age	− 0.20**	− 0.23**	− 0.13**	− 0.19**	− 0.18**	− 0.09**	0.10**
BMI	− 0.01	0.00	0.01	0.07	0.03	− 0.17**	− 0.07*
PF MVCt	0.14**	0.17**	0.10**	0.16**	0.07*	− 0.06*	− 0.07*
PF_RTD50_	0.14**	0.18**	0.09*	0.17**	0.19**	− 0.05*	− 0.21**
PF_RTD200_	0.16**	0.18**	0.12**	0.15**	0.09**	− 0.04	− 0.08*
DF MVCt	0.07*	0.06	0.08*	0.04	0.01	− 0.02	0.00
DF_RTD50_	0.09**	0.10**	0.08*	0.10**	0.14**	− 0.03	− 0.17**
DF_RTD200_	0.07*	0.05	0.09*	0.04	0.03	− 0.01	− 0.01
PF_RoM_	− 0.08*	− 0.07*	− 0.07*	− 0.06	0.13**	0.00	− 0.19**
DF_RoM_	− 0.04	− 0.01	− 0.06	− 0.03	0.07*	0.06*	− 0.15**

*Significant at p < 0.05.

**Significant at p < 0.01.

### Regression analysis

Age and gender significantly influenced all body sway variables except CoP frequency AP which was negatively influenced by BMI. Age significantly and negatively influenced all body sway variables, except CoP frequency ML which was positively influenced by age (first blocks, Table 4). Also, we found that all body sway variables were lower in women, except CoP frequency ML, which was higher.

**Table 4:** Sequential linear regression models of body sway variables.

Predictors	CoP velocity						CoP amplitude				CoP Frequency			
	Total		AP		ML		AP		ML		AP		ML	
	Block 1	Block 2	Block 1	Block 2	Block 1	Block 2	Block 1	Block 2	Block 1	Block 2	Block 1	Block 2	Block 1	Block 2
Age	− 0.19***	− 0.18***	− 0.23***	− 0.20***	− 0.12**	− 0.13**	− 0.20***	− 0.17***	− 0.18***	− 0.13**	− 0.04	− 0.06	0.13**	0.06
Gender	− 0.34***	− 0.37***	− 0.36***	− 0.40***	− 0.28***	− 0.31***	− 0.32***	− 0.37***	− 0.23***	− 0.33***	0.02	0.02	0.10**	0.19***
BMI	0.01	0.03	0.02	0.04	0.00	0.02	0.08*	0.09*	0.06	0.08	− 0.15**	− 0.12**	− 0.10*	− 0.09*
PF MVCt		− 0.13		− 0.04		− 0.20		0.02		− 0.07		− 0.19		− 0.08
PF_RTD50_		0.01		0.06		− 0.03		0.08		0.13*		− 0.09		− 0.15**
PF_RTD200_		0.12		0.06		0.17		− 0.02		0.00		0.16		0.12
DF MVCt		0.00		− 0.02		0.02		− 0.07		0.02		0.10		− 0.07
DF_RTD50_		0.02		0.03		0.02		0.03		0.12		0.02		− 0.21**
DF_RTD200_		− 0.07		− 0.10		− 0.03		− 0.08		− 0.16		− 0.03		− 0.27**
PF_RoM_		0.03		0.04		0.01		0.05		0.20***		− 0.04		− 0.22***
DF_RoM_		0.02		0.03		0.00		0.05		0.21***		− 0.03		− 0.25***
R^2^	0.15	0.16	0.18	0.20	0.10	0.10	0.15	0.17	0.09	0.19	0.03	0.05	0.03	0.17
adjusted R^2^	0.15	0.14	0.18	0.18	0.09	0.08	0.14	0.15	0.08	0.17	0.03	0.03	0.03	0.16
SEE	10.40	10.44	7.10	7.09	7.19	7.22	1.77	7.76	2.48	2.36	0.76	0.76	1.88	1.75
F	36.19***	9.37***	44.89***	12.12***	20.95***	5.53***	34.91***	9.92***	19.41***	11.34***	6.06***	2.35**	6.68***	10.20***

Values are standardized regression coefficients from linear regression analysis. 1 = males; 2 = females.

*AP* anterior–posterior, *ML* medial–lateral, *BMI* body mass index, *PF MVCt* maximal voluntary contraction torque for plantar flexion, *PF*_*RTD50*_ plantar flexion rate of torque development for 0–50 ms, *PF*_*RTD200*_ plantar flexion rate of torque development for 0–200 ms, *DF MVCt* maximal voluntary contraction torque for dorsal flexion, *DF*_*RTD50*_ dorsal flexion rate of torque development for 0–50 ms, *DF*_*RTD200*_ dorsal flexion rate of torque development for 0–200 ms, *PF*_*RoM*_ plantar flexion range of motion, *DF*_*RoM*_ dorsal flexion range of motion, *R*^*2*^ coefficient of determination, *F* F statistic, *SEE* standardized error of the estimate.

*Significant at p ≤ 0.05.

**Significant at p ≤ 0.01.

***Significant at p ≤ 0.001.

Ankle strength and RoM could not predict CoP velocity total (F_(9, 592)_ = 0.52, p = 0.86, R^2^ = 0.01), AP (F_(9, 592)_ = 1.16, p = 0.32, R^2^ = 0.01), ML (F_(9, 592)_ = 0.45, p = 0.91, R^2^ = 0.01), over and above age and gender alone. Measures of ankle strength and RoM did not also influence CoP amplitude AP (F_(9, 592)_ = 1.50, p = 0.14, R^2^ = 0.02) after accounting for significant age, gender, and BMI effects. However, PF_RTD50_ and PF_RoM_ and DF_RoM_ positively influenced CoP amplitude ML after controlling for significant age, gender, and BMI effects (F_(9, 592)_ = 7.97, p < 0.001, R^2^ = 0.10).

CoP frequency AP was not significantly fitted over and above BMI alone (F_(9, 592)_ = 1.11, p = 0.35, R^2^ = 0.02). CoP frequency ML was nevertheless fitted with ankle strength and RoM measures significantly better than age, gender, and BMI alone (F_(9, 592)_ = 11.04, p < 0.001, R^2^ = 0.14). We found the significant and negative impact of PF_RTD50_, DF_RTD50_, DF_RTD200_ and PF_RoM_ and DF_RoM_ on CoP frequency ML. Results of linear regressions of body sway variables using a hierarchical model building approach are presented in Table 4.

## Discussion

The purpose of this study was to determine if ankle RoM and strength variables were important predictors of body sway parameters during single-leg quiet stance in highly trained athletes from different sports. The most important result was that significant relationships were revealed between RoM measurement and CoP amplitude and frequency. Concerning the contribution of ankle strength in body sway variables, only PF_RTD50_ was significantly related to CoP amplitude (ML direction). Moreover, both, PF and DF strength were significant predictors for a CoP frequency in the frontal plane. There were no significant predictors for CoP velocity except for age and gender. Therefore, our hypothesis that ankle strength and RoM would be a predictor of postural stability during quiet stance was partially supported, having in mind that a very low amount of the variance in body sway outcomes was explained.

There is considerable evidence linking balance to the overall athlete’s strength^31^, injury risk^32^, but also to an athlete’s performance^27^. According to Kouzaki et al.^23^ age-related increases in postural sway are related to decreases in muscle volume. Moreover, research showed that the activity of the ankle PF during quiet standing is far smaller than the maximum^33,34^. However, most of the studies have used bilateral quiet standing for measuring postural sway. We have showed in the current study that PF strength (PF_RTD50_) was related to CoP amplitude in ML direction during single-leg quiet stance. Additionally, PF and DF strength were related to the CoP frequency in ML direction as well. However, it must be stated that only a small proportion of variance was explained (0–12%). The difference in results for ML and AP direction can be explained by the task requirements during upright standing where largest fluctuations occur in AP direction^35^. Different mechanisms for AP and ML plane postural control during quiet standing^36^ may contribute to above mentioned association. Accordingly, ML direction is controlled predominantly by frontal plane motion at the hip, whereas AP direction is under ankle control^37^. Moreover, Pellecchia^38^ showed that controlling AP direction requires greater attention compared to ML direction when task difficulty is increased in young healthy adults. Based on aforementioned, greater association between PF strength and CoP amplitude in AP direction should be expected, which was not the case in our study. Possible reasons for the difference in the results for AP and ML directions could be attributed to the fact that we used single-leg stance compared to other studies. Single-leg standing requires additional stabilization in ML direction, while several muscles of the ankle act in both frontal and sagittal planes. For instance, tibialis anterior, extensor hallucis longus and brevis as well as hallucis flexors and tibialis posterior are primarily acting as investors, while they also have a significant role in plantar and dorsal flexion. While this explains why we found associations between PF and DF strength with ML CoP, the reason for absence of associations in AP is less clear. We could speculate that under the single-leg conditions, the strength of the muscles becomes relevant only for the ML direction. One the other hand, postural sway in AP direction requires less frequent and less forceful muscle corrections.

Moreover, the current results regarding the ML directions corroborates the earlier research which found that control in bipedal standing is accomplished mainly by hip invertors and evertors, while one-leg standing requires fine frontal ankle control^39^. The fact that different control strategies are used during bipedal and unipedal standing has important implications for athletic performance and injury prevention, as adaptations to postural balance training have been reported to be highly task-specific^40^.

Smaller neuromuscular demands during quiet stance in elite athletes were confirmed by Sawers et al.^41^ and later by Kim et al.^42^ in non-ecological postural conditions. Sawers et al. (2015) observed less muscle co-activation and better muscle output efficiency in elite ballet dancers compared to novice dancers^41^. Similarly, Kim et al. (2018) found that elite female ice hockey players show lower co-activation of ankle PF and DF compared to non-athletes during unexpected external perturbations^42^. Slightly larger involvement of PF than DF strength in CoP amplitude during quiet stance was found in our highly trained athletes. Our findings concerning the association of ankle PF strength and CoP amplitude support the statement that static balance may be more related to PF strength than DF strength^43^.

The available literature on relationships between ankle RoM and static balance is limited in a population of healthy trained athletes. Bennell and Goldie^44^ found that a restriction of ankle RoM by external support reduces postural stability in young adults. This was confirmed by Kim and Kim^24^ who found that out of ankle RoM measures and various lower limb strength measures, ankle PF RoM in young adults showed the greatest association with static balance control ability. On the contrary, one study showed there was no relationship between ankle RoM and postural balance in younger adults^43^. In the present study, the PF_RoM_ and DF_RoM_ were correlated with CoP amplitude in ML direction and CoP frequency during quiet stance.

The discrepancy in the results concerning the association of RoM and body sway and the difference between ML and AP direction in the current study may be due to several reasons. The athletes in the current study were highly trained from different individual and team sports. Moreover, some athletes were younger, probably with incomplete postural control system maturation^45^. There is a greater average decrement of ankle joint RoM in females than males with aging^22^, which was confirmed in our study where age and gender were significant predictors in almost all body sway components. Additionally, most of the studies that investigated the association of ankle RoM and body sway were conducted on young adults, adults, as well as older adults, with little possibility of comparing our results with other results from similar studies. Nevertheless, the biggest strength of our study is a large sample of highly trained athletes from different sports. The results from the current study showed that body sway during quiet stance is similar in both legs regardless of a sport confirming that highly trained athletes use both limbs effectively in playing and stabilizing the body^46^. Additionally, the current study showed which control strategy during unipedal standing is used by highly trained athletes.

A few limitations should be mentioned, in particular in view of the nature of testing approach. Although single-leg standing tests are common in obtaining standardized data, it would be desirable for highly trained athletes to assess balance under more complex or unstable conditions. Another important limitation is the fact that participants were highly trained and healthy. Therefore, the results cannot be generalized to injured populations or older adults. Studies in which researchers would investigate similar relations in injured populations, are warranted. Additionally, Hertel et al.^47^ stated that foot morphology affects the postural sway in single-leg stance, which was not considered in the present study. Finally, based on a small proportion of the variance that explained body sway during single-leg quiet stance, it remains unclear whether balance and ankle strength and RoM are independent or dependent neuromuscular capacities in healthy young trained athletes. The findings from the current study provide further evidence concerning the relationship between increased ankle strength and RoM and body sway. However, due to the fact that only small amount of the variance in CoP variables was explained, further research are warranted.

## Conclusions

In the present study, we demonstrated that postural balance during single-leg stance in young trained athletes is correlated with ankle ROM and muscle strength. Specifically, ankle RoM showed significant relationship with CoP variables in ML direction. Accordingly, decreased ankle RoM can be considered as an important determinant of body sway in trained athletes. The results of the current study could be of benefit to the practitioners seeking to identify postural profile and to make an appropriate adjustment for balance performance enhancement.
